# Management of gallstone-induced severe acute cholecystitis and pancreatitis in the second trimester of pregnancy during covid-19 pandemic: A case report

**DOI:** 10.1016/j.amsu.2021.102563

**Published:** 2021-07-15

**Authors:** Adeodatus Yuda Handaya, Aditya Rifqi Fauzi, Joshua Andrew, Ahmad Shafa Hanif, Kevin Radinal, Azriel Farrel Kresna Aditya

**Affiliations:** a Digestive Surgery Division, Department of Surgery, Faculty of Medicine, Universitas Gadjah Mada/Dr. Sardjito Hospital, Yogyakarta 55281, Faculty of Medicine, Universitas Gadjah Mada/Dr Sardjito Hospital, Yogyakarta, Indonesia; b Faculty of Medicine, Universitas Gadjah Mada/Dr. Sardjito Hospital, Yogyakarta, 55281, Indonesia

**Keywords:** Acute cholecystitis, Acute pancreatitis, Cholelithiasis, Pregnancy, Covid-19 pandemic

## Abstract

**Introduction:**

Gallstone-induced severe acute cholecystitis with acute pancreatitis during pregnancy can be life-threatening both for the mother and fetus. Surgical approach is recommended in this complicated disease to prevent morbidity and mortality. During COVID-19 pandemic, additional precautions are needed when dealing with abdominal complaints.

**Presentation of case:**

We present a 37-year-old female patient, pregnant at 22 weeks gestational age, who complained of fever, diffuse abdominal pain, and shortness of breath. Laboratory examination results revealed anemia, leukocytosis and an increase in amylase level. SARS-CoV-2 antibody is non-reactive. Imaging strongly suggested cholelithiasis and cholecystitis. The patient was given antibiotics for three days but there was no significant improvement. Open cholecystectomy with subcostal (Kocher) incision was performed. Patient was released from the hospital without post-operative complications.

**Discussion:**

Treatment of gallstone induced severe acute cholecystitis with acute pancreatitis during pregnancy is challenging with the surgical complications. In the second and third trimester of pregnancy, it is more difficult to perform laparoscopic cholecystectomy because of the size of uterus. Laparoscopic procedure is also not recommended in early Covid-19 pandemic period. Therefore, open cholecystectomy with Kocher incision becomes the surgery of choice to avoid preterm birth.

**Conclusions:**

Based on our case, open cholecystectomy with Kocher incision is a safe and effective procedure for pregnant patients with cholelithiasis, cholecystitis, and pancreatitis.

## Highlights

• Gallstone induced acute severe cholecystitis and acute pancreatitis during pregnancy is a complicated disease.
• Cholecystitis and pancreatitis have been reported for producing similar symptoms to COVID-19 disease.
• This Case requiring immediate surgical management, advanced gestational age surgery may become a challenge.
• Open cholecystectomy with Kocher incision is a safe and effective for pregnancy with acute abdominal case.

## 1 Introduction

Cholelithiasis is a common occurrence during pregnancy due to increasing level of progesterone and estrogen. Increased estrogen level is often associated with the secretion of biliary cholesterol in the liver, prompting the accumulation of bile, which is high in cholesterol content. High level of estrogen and progesterone hormone also reduces the contractility of gallbladder smooth muscle which impaired the gallbladder from draining the accumulated biliary sludge out. As a result, there is a stasis of cholesterol-supersaturated bile which induces formation of gallstones. As progesterone and estrogen keep increasing proportionally with the duration of gestation, the incidence of gallstone formation becomes higher in the last two trimesters of pregnancy and hence cholelithiasis is especially very common during the third trimester [1].

Gallstones can lead to inflammation and subsequently infection of the gallbladder when it obstructs the flow of bile, resulting in cholecystitis. In rare cases, the gallstones can move further and obstruct the ducts close to the pancreas, which results in pancreatitis [2]. Acute pancreatitis in pregnancy is an uncommon condition with a varying incidence of approximately 1 in 1000–12,000 pregnant women and is considered an obstetrical emergency. Acute pancreatitis is often life-threatening to maternal and fetal health, resulting in a mortality rate of up to 37% for the mother and 60% for the fetus. Most pancreatitis in pregnancy is associated with gallstones as the main pathogenic cause [3].

In recent studies, the virus SARS-CoV-2 has been found to also cause abdominal symptoms such as vomiting, diarrhea, or abdominal pain, especially in the early phase of the infection. The infection results in the changes of intestinal microbes and inflammatory cytokines associated with the viral infection. Hence, it is important to be cautious of abdominal symptoms in this Covid-19 pandemic period especially when patient show respiratory symptoms as well [4].

Laparoscopic cholecystectomy is considered the recommended surgical method for cholecystitis in pregnancy. However, the decision to undergo open surgery is influenced by many factors, such as delayed diagnosis, the degree of severity of the disease or the complications present, or the surgeon's decision making. Technical difficulties of laparoscopic surgery during advanced stage of pregnancy also contributed to the decision to perform open cholecystectomy [5]. This work has been reported in line with the SCARE 2020 criteria [6].

## 2 Case presentation

A 37-year-old female patient, who is 22 weeks pregnant, came to the obstetrician with main complaints of fever, pain throughout the abdomen especially in the upper part, and shortness of breath. Following the procedural standard during the covid-19 pandemic, the patient was tested for SARS-Cov-2 IgM/IgG twice and both showed non-reactive results.

**Fig. 1:**
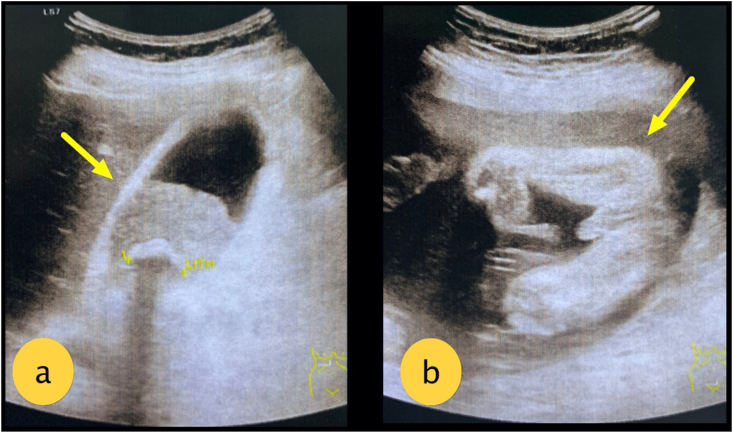
Abdominal USG: **(a)** Thickened gallbladder wall with an accumulation of fluid inside gallbladder lumen and posterior acoustic shadow suggesting of gallbladder inflammation and gallbladder stone; **(b)** Fetus located inside the uterus.

On the day of admission, routine blood examination showed hemoglobin level of 11.5 g/dL, leukocyte count of 19.400/μL, neutrophil percentage of 81.8%, and lymphocyte percentage of 12.0%. The patient was also subjected to ultrasonography (USG), which revealed multiple stone within the gallbladder and thickening of gallbladder wall suggesting of cholelithiasis, acute cholecystitis, gallbladder hydrops and Fetus located inside the uterus (Fig. 1). The patient was then given antibiotic treatment using 1 g of meropenem thrice daily for 3 days.

**Fig. 2:**
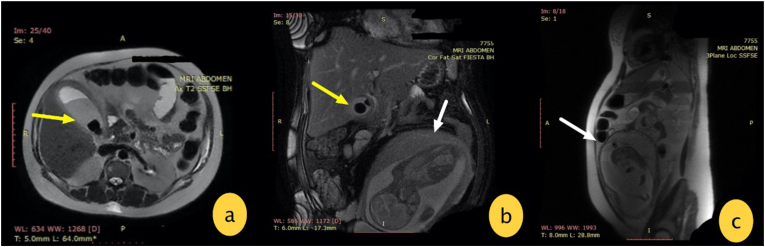
(a) axial view of the abdomen showing gallbladder dilatation with biliary sludge filling the lumen and multiple gallbladder stones; **(b)** coronal view of the abdomen showing contracted gallbladder with multiple gallbladder stones and enlarged uterus filled with fetus; **(c)** sagittal view of the abdomen showing presence of fetus in the uterus.

On the second day, patient had magnetic resonance imaging (MRI) and the result showed signs of cholelithiasis with multiple stones and biliary sludge filling the lumen and signs of acute cholecystitis with fetus in the uterus (Fig. 2). Patient's blood was taken for culture and antibiotic sensitivity testing which revealed meropenem-sensitive *Staphylococcus aureus*.

On the third day, patient claimed that the fever and pain had not significantly improved even at the end of antibiotic treatment. Laboratory examination showed hemoglobin level of 8.6 g/dL, leukocyte count of 19.340/μL, neutrophil percentage of 89.7%, and lymphocyte percentage of 5.1%. Bilirubin examination showed total bilirubin level of 0.38. Pancreatic enzymes test showed amylase level of 339 U/L, and lipase level of 8.8 U/L. Liver function test showed SGOT level of 7.8 U/L, SGPT level of 7.2 U/L, alkaline phosphatase level of 5.7 U/L, and albumin level of 2.9 g/dL. Based on this set of test results, we suspected that there was also an occurring hemorrhagic necrotizing pancreatitis due to the drop in hemoglobin level and increase in pancreatic enzymes level.

**Fig. 3:**
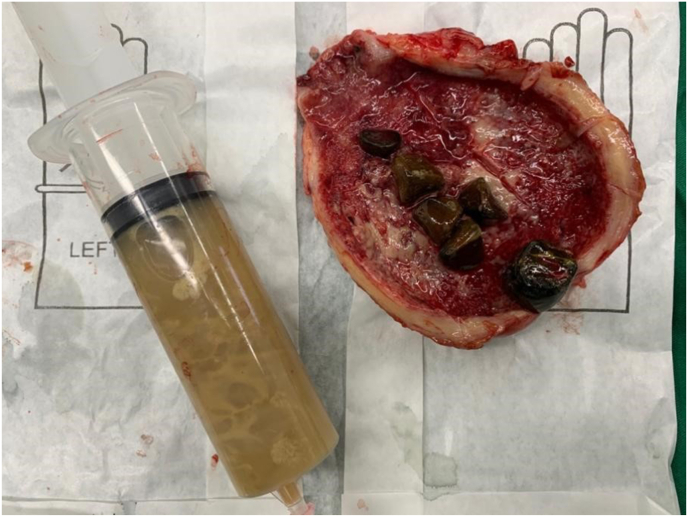
Post-operative finding: gallbladder with thickened wall, multiple gallbladder stones and gallbladder empyema.

On the fourth day, patient received blood transfusion and hemoglobin level increases to 13.9 g/dL and was allowed to undergo surgery. Patient underwent open cholecystectomy with subcostal incision (Kocher) performed by a senior gastrointestinal surgeon. Post-operative have found thickened wall of gallbladder with multiple gallbladder stones and gallbladder empyema (Fig. 3).

The patient was released from the hospital after 5 days of post-operative care. At the end of care, the patient claimed that the abdominal pain had been significantly reduced and free of fever. Routine check-ups were scheduled until the patient's delivery date. There was no further complaint by the patient during the follow-up period. In the end, the patient gave birth to the baby at term without any complications related to the cholecystitis and pancreatitis that she had previously experienced.

## 3 Discussion

We identified cholelithiasis, cholecystitis, and pancreatitis from imaging results and laboratory examinations. According to the Tokyo guidelines, the severity of cholecystitis in this patient is considered severe (grade 3) because the infection has spread to the pancreas. Therefore, it requires immediate surgical management to prevent further complications [7]. Conventionally, the clinical decision to choose between laparoscopic cholecystectomy (LC) or open cholecystectomy (OC) is determined by the patient's gestation. LC is associated with fewer fetal and maternal complications than open cholecystectomy in pregnant patients [5]. LC has many limitations, especially at a later gestational age where the uterus has filled a sizable abdominal cavity. Particularly in the third trimester, laparoscopic tools passing through the umbilical port can pose a large risk of uterine manipulation. Large uterus also hinders the movement of tools, resulting in poor vision and limitation of access for laparoscopic surgery [5].

From imaging results and observation made during surgery, pancreatitis is more likely to be caused by a spreading infection, which originates from the gallbladder. There is no evidence of any biliary stone obstruction close to the pancreatic area. We decided to perform OC with subcostal (Kocher) incision instead of the conventional median incision to prevent manipulation and stimulation of the fundus of the uterus that could result in preterm birth. Antibiotic therapy of 1gr meropenem thrice a day is also provided before surgery because meropenem is the gold standard drug for complicated abdominal infections and has been proven to be safe in pregnancy [8].

In cases of complicated abdominal infections, OC can become the more appropriate choice. Conversion to OC was done to anticipate grade 2 or 3 adhesion of gallbladder (usually referred as ‘difficult gallbladder’) and the surrounding omentum as a result of an extensive cholecystitis. Furthermore, the surgeon believed that performing OC in this patient will result in quicker procedure after considering all of the patient's condition: pregnant at second trimester, possible gallbladder adhesion, and suspected pancreatitis. This situation is reported in a review article about factors predisposing conversion from LC to OC in which ‘difficult gallbladder’ become one of the many reasons for conversion. The conversion rate from LC to OC for ‘difficult gallbladder’ in non-pregnant patients was 22% [9]. According to a systematic review by Sedaghat et al., the conversion rate from LC to OC in pregnant patients with symptomatic cholelithiasis was 3.8% [5].However, conversion rate from LC to OC in pregnant patients with ‘difficult gallbladder’ and pancreatitis has not been reported yet.

In this Covid-19 pandemic period, additional precautions must be taken when dealing with patients. Many intra-abdominal diseases such as cholecystitis and pancreatitis have also been reported for producing similar symptoms to COVID-19 symptoms such as fever, dyspnea, and cough [9]. Meanwhile, abdominal pain which is present in this patient is in fact a typical symptom of COVID-19. Respiratory symptoms usually appear in the late phase while abdominal symptoms are often present in the early phase of the disease. It has been reported that the virus can bind with Angiotensin-converting enzyme 2 (ACE2) receptors in the intestine, which makes the intestine susceptible to inflammation resulting in common symptoms such as diarrhea, nausea, vomiting, anorexia, or abdominal pain. Therefore, surgeries for intra-abdominal diseases require strict screening and tight protocol to minimize the risk of transmission of COVID-19 [4,10].

Additionally, the patients had their surgery shortly after Indonesian Society of Digestive Surgeons released a guideline in the year of 2020 to address recommended surgical approach amid early COVID-19 pandemic. One of the recommendation states that surgeon should perform a surgery that has the least operative time and avoiding laparoscopic procedure. The rationale behind this recommendation is to minimize contact time between surgeon and patients, as well as minimizing the use of surgical procedure that utilizes gas and plumes. Aerosol generating procedures done in operating theatre poses very high risk of viral transmission [11].

## 4 Conclusion

This case report highlights the use of open cholecystectomy with Kocher incision in pregnant patients. The conversion from LC to OC in this case was influenced by many factors, and therefore we believed OC will pose less risk of preterm birth as compared to LC. OC also allows better exploration when anticipating other intra-abdominal diseases that needs to be treated immediately. In conclusion, in pregnant patients with gallstone induced severe acute cholecystitis with concurrent acute pancreatitis, open cholecystectomy with subcostal (Kocher) incision can become the treatment of choice because it is safe and effective.

## Provenance and peer review

Not commissioned, externally peer-reviewed.

## Declaration of competing interest

No potential conflict of interest relevant to this article was reported.

## Registration of research studies

The manuscript is a case report, not considered a formal research involving participants.
